# Could the HALP score serve as a biomarker of bronchiectasis exacerbation?

**DOI:** 10.5578/tt.202401811

**Published:** 2024-03-26

**Authors:** Mustafa ÇOLAK, Hikmet ÇOBAN, Nurhan SARIOĞLU, Merve YUMRUKUZ ŞENEL, Fuat EREL

**Affiliations:** 1 Department of Chest Diseases, Balıkesir University Faculty of Medicine, Balıkesir, Türkiye

## Abstract

**ABSTRACT**

**
Could the HALP score serve as a biomarker of bronchiectasis exacerbation?
**

**Introduction:** *Bronchiectasis is a chronic inflammatory lung disease and patients may occasionally experience acute exacerbations. Our study aims to determine the relationship between exacerbation periods and HALP (hemo- globin, albumin, lymphocyte, platelet) scores in patients with bronchiectasis.
*

**Materials and Methods:** *Adult patients diagnosed with bronchiectasis and followed up in our clinic between 02.2020-12.2022 were retrospectively evaluated. After the examinations, the effect of bronchiectasis exacerbation on the HALP score was investigated.
*

**Results:** *A total of 84 patients diagnosed with non-cystic fibrosis bronchiecta- sis were included in our study. 42 of the patients were male (50%), and 42 were female. The average age of all patients was 52.37 ± 16.2. 35 patients (41.7%) were in the exacerbation period, and 49 patients (58.3%) were in the stable period. The median values of leukocytes, neutrophils, and C-reactive protein (CRP) were significantly higher in patients during the exacerbation period compared to the stable period (respectively p= 0.00, p= 0.00, p= 0.00). The average values of FEV1% and FVC% in patients during the exacer- bation period were significantly lower compared to the stable period (p= 0.03, p= 0.00, respectively). The HALP score was significantly lower in patients during the exacerbation period compared to the stable period (p= 0.00). A significant negative correlation was found between the HALP score and leukocytes, neutrophils, and CRP (p= 0.00, p= 0.00, p= 0.00, respec- tively). Also, a significant positive correlation was found between the HALP score and FEV1% and FVC% (p= 0.00, p= 0.00, respectively).
*

**Conclusion:** *Our study revealed that the HALP score is associated with infec- tious and pulmonary functional parameters in bronchiectasis patients in the exacerbation period. We propose that the HALP score could serve as a valu- able biomarker during exacerbations.
*

**Key words:** *Bronchiectasis; halp score; exacerbation
*

**ÖZ**

**
HALP skoru bronşektazi alevlenmesinin biyolojik belirteci olabilir mi?
**

**Giriş:** *Bronşektazi kronik enflamatuvar bir akciğer hastalığıdır ve hastalık süre-
*

*
cinde ara sıra akut alevlenmeler yaşanabilmektedir. Çalışmamız bronşektazi
*

*
hastalarında alevlenme dönemi ile HALP skoru (hemoglobin, albümin, lenfosit, trombosit) arasındaki ilişkinin belirlenmesini amaçla- maktadır.
*

**Materyal ve Metod:** *Kliniğimizde bronşektazi tanısı ile 02.2020-12.2022 tarihleri arasında takip edilen erişkin hastalar retrospektif olarak değerlendirildi. Yapılan incelemeler sonrasında bronşektazi alevlenmesinin HALP skoru üzerine etkisi araştırıldı.
*

**Bulgular:** *Çalışmamıza kistik fibrozis dışı bronşektazi tanılı 84 hasta dahil edildi. Hastaların 42’si (%50) erkek, 42’si kadın idi. Tüm hastaların yaş ortalamaları 52,37 ± 16,2 idi. Otuz beş hasta (%41,7) alevlenme döneminde, 49 hasta (%58,3) stabil dönemdeydi. Lökosit, nötrofil ve C-reaktif protein (CRP) medyan değerleri, alevlenme dönemindeki hastalarda stabil döneme göre anlamlı ölçüde daha yüksek saptandı (sırasıyla p= 0,00, p= 0,00, p= 0,00). Alevlenme dönemindeki hastaların FEV1% ve FVC% ortalama değerleri, stabil döneme göre anlamlı ölçüde daha düşük saptandı (sırasıyla p= 0,03, p= 0,00). HALP skoru, alevlenme dönemindeki hastalarda, stabil döneme göre anlamlı derecede daha düşük saptandı (p= 0,00). HALP skoru ile lökosit, nötrofil ve CRP arasında negatif yönde anlamlı ilişki saptandı (sırasıyla p= 0,00, p= 0,00, p= 0,00). HALP skoru ile FEV1% ve FVC% arasında pozitif yönde anlamlı ilişki saptandı (sırasıyla p= 0,00, p= 0,00).
*

**Sonuç:** *Çalışmamız sonucunda, HALP skorunun alevlenme döneminde olan bronşektazi hastalarında enfeksiyöz ve pulmoner fonksi- yonel parametreler ile ilişkili olduğunu saptadık. HALP skorunun alevlenme döneminde bir biyobelirteç olarak kullanılabileceğini düşünüyoruz.
*

**Anahtar kelimeler:** *Bronşektazi; halp skoru; alevlenme
*

## INTRODUCTION

Bronchiectasis is the abnormal and permanent widening of the bronchi. It is often accompanied by chronic productive cough, airway obstruction, and recurrent infections. Bronchiectasis is not a single disease but a result arising from a variety of causes (1).

The first descriptions related to bronchiectasis were proposed by Laennec in 1819 (2). In the 1950s, Reid combined bronchography findings with pathology evaluations and divided bronchiectasis into three phenotypes: tubular, varicose, and cystic (3).

The exact prevalence of bronchiectasis is unknown. It is thought to be more common in regions where access to healthcare is limited and lower respiratory tract infections are common during childhood. With the introduction of antibiotics, the frequency of bronchiectasis is accepted to have decreased compared to the pre-antibiotic period (3,4). Bronchiectasis is an age-related disease (5). An increased incidence of serious disease is observed in the elderly (6). The prevalence of bronchiectasis varies by region and its exact prevalence is unknown. The prevalence of bronchiectasis in Germany was 67/100.000 between 2005-2011 (7). In Catalonia, a large population-based study similarly found a high prevalence of 36.2/10000 (8). The overall prevalence of bronchiectasis in the United States is approximately 139 cases per 100.000 adults aged 18 and over (9).

Following anatomical damage in the bronchi, exacerbations tend to increase due to the progressive deterioration of pulmonary physiology (3). Bronchiectasis is a heterogeneous disease. This heterogeneity is present both in the stable state and

during exacerbations. A common definition of exacerbation was developed for clinical trials in 2016. An exacerbation was defined as a change associated with worsening of three or more of the following core symptoms over at least 48 hours in the treatment of bronchiectasis: cough; sputum volume and/or density; sputum purulence; shortness of breath and/or exercise tolerance; tiredness and/or malaise or hemoptysis (10).

Exacerbations are often triggered by bacterial agents, and guidelines recommend antibiotic therapy for management (11,12). Bronchiectasis is a chronic inflammatory disease in which airway inflammation is predominantly neutrophil-dominated (13). In Cole’s hypothesis, the airway is an important component of inflammation (14). It is known that patients with bronchiectasis show a systemic inflammatory response (15). Saleh et al. showed a correlation between systemic inflammation and severity of bronchiectasis in 87 patients (16).

The hemoglobin, albumin, lymphocyte, and platelet (HALP) score is an indicator of nutritional status and systemic inflammation. HALP score is determined by the following formula: hemoglobin (g/L) x albumin (g/L) x lymphocytes (/L)/platelet (/L) (17). The HALP score is a comprehensive index that measures immune health as well as nutritional status (18). To date, there have been no studies in the literature examining the relationship between the HALP score and acute exacerbations of bronchiectasis.

Our study aims to examine the potential utility of the HALP score as a biomarker during bronchiectasis exacerbation and its correlation with infectious parameters.

## MATERIALS and METHODS

This study was approved by the ethics committee and conducted in full accordance with the guidelines of the Declaration of Helsinki. The requirement for informed consent from the patients was waived due to the retrospective nature of the study.

Cases aged 18 and over, who were followed up with a diagnosis of non-cystic fibrosis bronchiectasis in the chest diseases clinic between February 2020 and December 2022, were evaluated retrospectively. Repeated applications of cases were not included in the study. Exclusion criteria included cancer, chronic inflammatory disease, hematological disease, and severe liver, and kidney failure. Two patient groups were formed, those in the stable period and those in the acute exacerbation period. Patient demographics, comorbidities, hemogram parameters, biochemical parameters (albumin, C-reactive protein), respiratory function tests, and radiological examinations were extracted from the online registration system. The change in the HALP score during the exacerbation and stable periods of the patients and the relationship of this score with infectious parameters were evaluated.

## Statistical Analysis

SPSS version 23.0 was used in the analysis. Continuous variables were reported as mean ± standard deviation in the study groups. Categorical variables were indicated as frequencies and percentages. The normality distribution of all data was performed by the Kolmogorov-Smirnov test. The Chi-square test was used for the evaluation of categorical data. Pearson’s *χ*2 or Fisher’s exact tests were utilized to compare categorical data. To decipher the relationship between the quantitative variables, correlation analysis was performed. While

the Pearson correlation results were taken as the basis for those variables that conform to the normality assumption, Spearman correlation analysis results were taken as the basis for variables that do not conform to the normality assumption. When the continuous data did not match the normal distribution, the Deciency between the binary groups was Mann- Whitney U, and when it matched the normal distribution, the Independent Samples t-test was performed. A significance level of 0.05 (*α*= 0.05) was used to determine statistical significance.

## RESULTS

84 patients diagnosed with non-cystic fibrosis bronchiectasis were included in our study. 42 of the patients (50%) were male, and 42 were female. The average age was 52.37 ± 16.2. 35 patients (41.7%) were observed during the exacerbation period, and 49 patients (58.3%) during the stable period. Half of all patients (n= 42) had comorbidities.

The median ages were found to be similar in the exacerbation and stable patient groups (p= 0.2). Comorbidities were present in 46.9% of the patients in the stable period and 54.2% of the patients in the exacerbation period, and no significant difference was found between the groups (p= 0.5) (Table 1). When evaluating comorbidities, we found that 10 patients had an endocrine system disease, 12 had hypertension, seven had heart disease, and four had a history of neurological disease.

Six of the patients in the stable period had asthma and one had COPD. Four of the patients in the exacerbation period had asthma and four had COPD. For the history of asthma and COPD, patients in the stable and exacerbation periods were observed similarly (respectively p= 0.9 and p= 0.07).

**Table:** 

**Table 1.** Demographic features and functional parameters of the patient groups			
	**Stable period (n= 49)**	**Exacerbation (n= 35)**	**p**
Age, years			
Median (min-max)	55 (22-73)	55 (24-87)	0.2
Gender, Male/Female	20/29	22/13	0.046
Comorbidity	23 (46.9%)	19 (54.2%)	0.5
FEV1%	67.07 ± 24.74	51.95 ± 23.15	0.03
FVC%	77.29 ± 20.16	57.68 ± 20.63	<0.001
FEV1/FVC	70.63 ± 13.29	71.9 ± 13.93	0.74
FEV1: Forced expiratory flow rate, FVC: Forced vital capacity.			

**Table:** 

**Table 2.** Biochemical features of patients in the stable and exacerbation periods			
	**Stable period (n= 49)**	**Exacerbation (n= 35)**	**p**
Neutrophil x103/µL median (min-max)	4.30 (2.0-11.8)	8 (2.7-25.8)	<0.001
Leukocyte x103/µL median (min-max)	7.5 (4-14)	10.6 (4.5-27.4)	<0.001
Monocyte x103/µL median (min-max)	0.6 (0.2-1)	0.7 (0.1-6.5)	0.36
C-reactive protein mg/L median (min-max)	3.20 (2.9-14.5)	42.9 (8-193)	<0.001
Albumin g/L median (min-max)	44 (30-49)	34 (25-44)	<0.001
HALP score	4.60 ± 2.25	2.35 ± 1.63	<0.001
HALP: Hemoglobin, albumin, lymphocyte, and platelet.

The average FEV1% and FVC% values in patients during the exacerbation period were significantly lower compared to those in the stable period (respectively p= 0.03, p= 0.00). The groups were similar in terms of FEV1/FVC values (p= 0.74) (Table 1).

The median leukocyte, neutrophil, and C-reactive protein (CRP) values were significantly higher in patients during the exacerbation period compared to those in the stable period (respectively p= 0.00, p= 0.00, p= 0.00). Monocyte values were similar between the groups (p= 0.36) (Table 2).

The median albumin values were significantly lower in patients during the exacerbation period compared to those in the stable period (p= 0.00). The average HALP score was significantly lower in patients during the exacerbation period compared to those in the stable period (p= 0.00).

Sputum culture yielded positive results in 15 patients (42.85%) during the exacerbation period. Culture results are shown in Table 3. There was no difference in the HALP score between patients with and without positive culture results (p= 0.84).

A significant negative correlation was found between the HALP score and leukocytes, neutrophils, and CRP (respectively p= 0.00, p= 0.00, p= 0.00). No significant relationship was found between the HALP score and monocytes (p= 0.74) (Table 4).

A significant positive correlation was found between

the HALP score and FEV1% and FVC% (respectively p= 0.00, p= 0.00) (Table 4).

A significant negative correlation was found between the HALP score and albumin and the prevalence of bronchiectasis (number of affected lobes) (respectively p= 0.00, r= -0.33, p= 00, r= -0.025).

**Table:** 

**Table 3.** Sputum culture results of patients during the exa- cerbation period		
**n, %**		
*Pseudomonas aeruginosa*	11	(31.42)
*Klebsiella pneumoniae*	2	(5.71)
*Acinetobacter baumannii*	1	(2.85)
*Staphylococcus aureus*	1	(2.85)
Non-cultivating	20	(57.14)

**Table:** 

**Table 4.** Relationship between the HALP score and functio- nal and biochemical parameters		
**HALP score**		
	**r**	**p**
Leukocyte	-0.31	<0.001
Neutrophil	-0.42	<0.001
Monocyte	0.03	0.74
C-reactive protein	-0.39	<0.001
FEV1%	0.50	<0.001
FVC%	0.52	<0.001
FEV1/FVC	0.22	0.12
HALP: Hemoglobin, albumin, lymphocyte, and platelet, FEV1: Forced expiratory flow rate, FVC: Forced vital capacity.		

## DISCUSSION

Bronchiectasis is a lung disease characterized by airway infection and inflammation that causes permanent damage to the small airways. Bronchiectasis can occur at all ages, but the highest disease prevalence is seen in the older age range (above 60) (19). In a meta-analysis in 2020 that included 6525 patients, the average age of cases was between 48.2 ± 16 and 68 ± 14.6 (20). The mean age of all patients participating in our study was 52.37 ± 16.2, consistent with the literature.

In patients with bronchiectasis, lung function impairment can be heterogeneous. While obstructive disorders are most commonly observed in respiratory function tests, mixed-type and restrictive-type disorders can also occur (21). During bronchiectasis exacerbations, a significant decrease is observed in FVC% and FEV1% values (22). In our study, the FEV1% and FVC% values of patients in the exacerbation group were significantly lower than patients in the stable period.

Systemic inflammation, characterized by elevated blood neutrophil levels, increased CRP, and plasma cytokines, has been reported in patients with bronchiectasis and is associated with disease severity (23,24). Similarly, in our study, we found that during the exacerbation period, when the disease was more severe, patients exhibited significantly higher levels of neutrophils, leukocytes, and CRP compared to the stable period.

While anemia and thrombosis can exacerbate inflammation, lymphocytes decrease inflammation (25). There are studies suggesting that albumin reflects inflammation and disease severity in acute illnesses (26). The HALP score, derived from the combination of these parameters, serves as a measure to assess inflammation-nutrition status. This finding can help clinicians assess prognosis and arrange appropriate treatments (17). Studies on cancer and stroke patients have concluded that a low HALP score is indicative of poor prognosis (17,18,27). In our study, we observed a significant decrease in the HALP score during disease exacerbation compared to the stable period, indicating its association with infectious parameters as an inflammation marker. This result suggests that the HALP score could serve as a valuable biomarker to guide clinicians in managing patients during exacerbation periods.

In a study involving 87 patients, significant relationships were reported between systemic inflammation assessed with leukocyte count, CRP, and erythrocyte sedimentation rate analyses, and the severity of bronchiectasis (15). The study by Wilson et al. supports the existence of a relationship between lung function and systemic inflammation in bronchiectasis (16). In our study, a significant relationship was found between the HALP score, which is a marker of systemic inflammation, and lung

functions (FEV1%, FVC%). We observed a correlation between low HALP scores and reduced lung function

in patients.

Gale et al. reported a decrease in albumin levels in patients with bronchiectasis compared to controls (28). In a study evaluating 177 patients with bronchiectasis in 2021, low albumin levels were found significant in predicting the severity of the disease and hospital admission (29). Similarly, in our study, albumin levels of patients in the exacerbation period were found to be lower than patients in the stable period.

In the study by Ju et al., it was found that in patients with bronchiectasis, there were more affected lobes in the patient group with low albumin levels compared to the normal albumin group (29). In our study, we observed a significant negative relationship between albumin levels, HALP score, and the number of affected lobes.

One limitation of our study was the small sample size, which may have affected the statistical power to detect significant effects. Secondly, our study was retrospective and observational, introducing the possibility of unmeasured confounding factors influencing our results. Therefore, multicenter cohort studies are warranted to validate our findings.

## CONCLUSION

In conclusion, our study revealed a decline in the HALP score during exacerbation periods in patients with bronchiectasis, and this decline was associated with infectious and pulmonary functional parameters. We believe that the HALP score should be included as a biomarker in the evaluation of patients during exacerbation periods.

**Ethical Committee Approval:** This study was approved by the Balıkesir University Clinical Research Ethics Committee (31/05/2023-91).

## CONFLICT of INTEREST

The authors declare that they have no conflict of interest.

## AUTHORSHIP CONTRIBUTIONS

Concept/Design: All of authors Analysis/Interpretation: MÇ, HÇ, NS Data acqusition: MÇ, MYŞ

Writing: MÇ, NS

Clinical Revision: MÇ, NS, FE Final Approval: All of authors

## REFERENCES

1. *
  Baydarian M, Walter RN. Bronchiectasis: Introduction, etiology and clinical features. Dis Mon 2008; 54: 516-26. https://doi.org/10.1016/j.disamonth.2008.05.007*
2. *
  Laennec RTH. De l’ Auscultation Mediate ou Traite du diagnostic des maladies des Poumons et du Coeur. (On Medite Auscultation or Treatise on the Diagnosis of the Diseases of the Lungs and Heart). Paris: Brosson and Chaude, 1819.
  *
3. *
  Barker AF. Bronchiectasis. N Engl J Med 2002; 346: 1383- 93. https://doi.org/10.1056/NEJMra012519*
4. *
  Bilton D, Jones AL. Bronchiectasis: Epidemiology and causes. Eur Respir Mon 2011; 52: 1-10. https://doi. org/10.1183/1025448x.10003110*
5. *
  Lonni S, Chalmers JD, Goeminne PC, McDonnell MJ, Dimakou K, Soyza AD, et al. Etiology of non-cystic fibrosis bronchiectasis in adults and its correlation to disease severity. Ann Am Thorac Soc 2015; 12: 1764-70. https:// doi.org/10.1513/AnnalsATS.201507-472OC*
6. *
  Quint JK, Millett ER, Joshi M, Joshi M, Navaratnam V, Thomas SL, et al. Changes in the incidence, prevalence and mortality of bronchiectasis in the UK from 2004 to 2013: A population-based cohort study. Eur Respir J 2016; 47(1): 186-93.https://doi.org/10.1183/13993003.01033- 2015*
7. *
  Ringshausen FC, de Roux A, Diel R, Hohmann D, Welte T, Rademacher J. Bronchiectasis in Germany: A popula- tion-based estimation of disease prevalence. Eur Respir J 2015; 46(6): 1805-7. https://doi. org/10.1183/13993003.00954-2015*
8. *
  Monteagudo M, Rodriguez-Blanco T, Barrecheguren M, Simonet P, Miravitlles M. Prevalence and incidence of bronchiectasis in Catalonia, Spain: A population- based study. Respir Med 2016; 121: 26-31. https://doi. org/10.1016/j.rmed.2016.10.014*
9. *
  Gramegna A, Pasquale MD, Blasi F. In: Chalmers J, Polverino E, Aliberti S, eds. Bronchiectasis: the EMBARC Manual. Cham, Switzerland: Springer International Publishing. 2018: 65-76.
  *
10. *
  Hill AT, Haworth CS, Aliberti S, Barker A, Blasi F, Boersma W, et al. Pulmonary exacerbation in adults with bronchi- ectasis: A consensus definition for clinical research. Eur Respir J 2017; 49: 1700051. https://doi. org/10.1183/13993003.00051-2017*
11. *
  Polverino E, Goeminne PC, McDonnell MJ, Aliberti S, Marshall SE, Loebinger MR, et al. European Respiratory Society guidelines for the management of adult bronchi- ectasis. Eur Respir J 2017; 50: 1700629. https://doi. org/10.1183/13993003.00629-2017*
12. *
  Hill AT, Welham SA, Sullivan AL. Updated BTS adult bron- chiectasis guideline 2018: A multidisciplinary approach to comprehensive care. Thorax 2019; 74: 1-3. https://doi. org/10.1136/thoraxjnl-2018-212468*
13. *
  Gramegna A, Amati F, Terranova L, Sotgiu G, Tarsia P, Miglietta D, et al. Neutrophil elastase in bronchiectasis. Respir Res 2017; 18: 211. https://doi.org/10.1186/ s12931-017-0691-x*
14. *
  Cole PJ. Inflammation: A two-edged sword-the model of bronchiectasis. Eur J Respir Dis Suppl 1986; 147: 6-15.
  *
15. *
  Wilson CB, Jones PW, O’Leary CJ, Hansell DM, Dowling RB, Cole PJ, et al. Systemic markers of inflammation in stable bronchiectasis. Eur Respir J 1998; 12: 820-4. https://doi.org/10.1183/09031936.98.12040820*
16. *
  Saleh AD, Chalmers JD, De Soyza A, Fardon TC, Koustas SO, Scott J, et al. The heterogeneity of systemic inflamma- tion in bronchiectasis. Respir Med 2017; 127: 33-9. https://doi.org/10.1016/j.rmed.2017.04.009*
17. *
  Tian M, Li Y, Wang X, Tian X, Pei LL, Wang X, et al. The hemoglobin, albumin, Lymphocyte, and platelet (HALP) score is associated with poor outcome of acute ischemic stroke. Front Neurol 2021; 11: 610318. https://doi. org/10.3389/fneur.2020.610318*
18. *
  Zhai B, Chen J, Wu J, Yang L, Guo X, Shao J, et al. Predictive value of the hemoglobin, albumin, lymphocyte, and plate- let (HALP) score and lymphocyte-to-monocyte ratio (LMR) in patients with non-small cell lung cancer after radical lung cancer surgery. Ann Transl Med 2021; 9(12): 976. https://doi.org/10.21037/atm-21-2120*
19. *
  O’Donnell AE. Bronchiectasis update. Curr Opin Infect Dis 2018; 31(2): 194-8. https://doi.org/10.1097/ QCO.0000000000000445*
20. *
  He M, Zhu M, Wang C, Wu Z, Xiong X, Wu H, et al. Prognostic performance of the FACED score and bronchi- ectasis severity index in bronchiectasis: A systematic review and meta-analysis. Biosci Rep 2020; 40(10): BSR20194514. https://doi.org/10.1042/BSR20194514*
21. *
  Yıldız Ö. Solunum fonksiyon testleri ve egzersiz testleri. In: Müsellim B, Karadağ B (eds). Bronşektazi-Toraks Kitapları, Ankara; 2015: 98-107.
  *
22. *
  Guan WJ, Gao YH, Xu G, Lin ZY, Tang Y, Li HM, et al. Characterization of lung function impairment in adults with bronchiectasis. PLoS One 2014; 9(11): e113373. https://doi.org/10.1371/journal.pone.0113373*
23. *
  Coban H, Gungen AC. Is there a correlation between new scoring systems and systemic inflammation in stable bron- chiectasis? Can Respir J 2017; 2017: 9874068. https:// doi.org/10.1155/2017/9874068*
24. *
  Dente FL, Bilotta M, Bartoli ML, Bacci E, Cianchetti S, Latorre M, et al. Neutrophilic bronchial inflammation cor- relates with clinical and functional findings in patients with noncystic fibrosis bronchiectasis. Mediators Inflamm 2015; 2015: 642503. https://doi.org/10.1155/2015/642503*
25. *
  Barlas RS, Honney K, Loke YK, McCall SJ, Bettencourt-Silva JH, Clarc AB, et al. Impact of hemoglobin levels and ane- mia on mortality in acute stroke: Analysis of UK regional registry data, systematic review, and meta-analysis. J Am Heart Assoc 2016; 5(8): e003019. https://doi. org/10.1161/JAHA.115.003019*
26. *
  Eckart A, Struja T, Kutz A, Baumgartner A, Baumgartner T, Zurfluh S, et al. Relationship of nutritional status, inflam- mation, and serum albumin levels during acute illness: A prospective study. Am J Med 2020; 133(6): 713-22.e7. https://doi.org/10.1016/j.amjmed.2019.10.031*
27. *
  Wei S, Shao J, Wang J, Wang G. The preoperative hemo- globin, albumin, lymphocyte, and platelet score is a prog- nostic factor for non-small cell lung cancer patients under- going adjuvant chemotherapy: A retrospective study. Ann Transl Med 2022; 10(8): 457. https://doi.org/10.21037/ atm-22-1097*
28. *
  Gale NS, Bolton CE, Duckers JM, Enright S, Cockcroft JR, Shale DJ. Systemic comorbidities in bronchiectasis. Chron Respir Dis 2012; 9(4): 231-8. https://doi. org/10.1177/1479972312459973*
29. *
  Ju S, Jeong JH, Heo M, Heo IR, Kim TH, Kim HC, et al. Serum albumin is a predictor of respiratory hospitaliza- tion in patients with bronchiectasis. Chron Respir Dis 2021; 18: 14799731211017548. https://doi. org/10.1177/14799731211017548*

